# Academic detailers’ and general practitioners’ views and experiences of their academic detailing visits to improve the quality of analgesic use: process evaluation alongside a pragmatic cluster randomized controlled trial

**DOI:** 10.1186/s12913-017-2797-8

**Published:** 2017-12-21

**Authors:** Sibyl Anthierens, Veronique Verhoeven, Olivier Schmitz, Samuel Coenen

**Affiliations:** 1 Department of Primary and Interdisciplinary Care (ELIZA), University of Antwerp, Campus Drie Eiken, Universiteitsplein 1, 2610 Antwerp, Belgium; 2 Department of Epidemiology and Social Medicine (ESOC), University of Antwerp, Antwerp, Belgium; 3 Research Institute Health and Society (IRSS), Catholic University of Leuven, Brussels, Belgium

**Keywords:** Continuing medical education, Educational outreach visits, Nonsteroidal anti-inflammatory drugs, Primary care, Prescribing behavior, Qualitative research, Interviews

## Abstract

**Background:**

Continuous medical education strategies, including academic detailing (AD), have mixed effects on the quality of prescribing in general practice. Alongside a cluster-randomized controlled trial (cRCT) to assess the effectiveness of AD visits (on appropriate prescribing of analgesics for chronic pain in osteoarthritis) by Farmaka, an independent drug information center, we performed a process evaluation to identify possible barriers and success factors to improve these AD visits, both from the perspective of the academic detailers delivering the visits and the general practitioners (GPs) receiving them.

**Methods:**

We performed semi-structured interviews with 20 GPs who participated in the cRCT and 13 academic detailers. The interviews were transcribed verbatim and analysed using thematic analysis.

**Results:**

GPs viewed AD visits as a practical and useful CME strategy, that is less time consuming than other CME activities, and the visitors as providers of objective and independent information relevant to their daily practice with whom they can have meaningful discussion. Academic detailers saw themselves as content experts, mainly informing GPs about the topic and not emphasizing on behavior change. Both GPs and academic detailers believed that the AD visits could have better interaction and discussion if performed in small groups. According to the GPs, the visits on analgesic use provided some new and relevant information as well as clarifying some misconceptions. They increased awareness of the disadvantages of particular non-steroidal anti-inflammatory drugs and of the lower doses of paracetamol that should be prescribed for chronic use, which may have changed their beliefs and/or attitudes towards more appropriate prescribing for osteoarthritis. However, the transfer of knowledge into practice was seen as not so straightforward.

**Conclusions:**

GPs view AD visits as a credible and interesting way of CME that enhances their knowledge and increases reflection on their prescribing behavior.

## Background

Understanding professional behavior change, including barriers and facilitating factors, is essential for clinicians and policy makers who seek to translate clinical evidence into practice. In primary care, interventions that are most effective in clinical trial settings may not necessarily be those that general practitioners (GPs) prefer to learn, find easiest to use, prioritize to implement or are most suitable for their practice environment [1]. Continuing medical education (CME) aims to support GPs to this end. In Western countries, over half of the CME strategies for GPs are supported by the pharmaceutical industry, biasing the information delivered [2]. Most CME strategies (e.g. educational materials, conferences, courses) have mixed effects, but academic detailing (AD) is especially effective for prescribing and prevention in general practice [3–8].

In Belgium, Farmaka (https://www.farmaka.be/nl) is an independent drug information center that has been active since the 1990s [9–11]. It has been operating a nationwide AD service (ADS) in Belgium since 2006 to contribute to the rational prescribing of medicines.

Farmaka applies the principles described by Soumerai and Avorn for AD, including the social marketing approach [12], as well as the principles of evidence-based medicine (EBM) [13, 14].

The effect of ADS to improve prescribing in general practice has only been researched in two small scale randomized controlled trials (RCTs) with GPs. The GPs did not have a longstanding relation with the academic detailers [9, 11]. Both RCTs are included in the relevant Cochrane review [2]. Another small RCT with factorial design (results of academic detailing have not been published) and a recent study funded by the Belgian Health Care Knowledge Centre (KCE) could not demonstrate its effectiveness [15]. The latter study however acknowledged serious limitations. Consequently we could not draw conclusions about the effectiveness of Farmaka’s ADS. Therefore, the Federal Agency for Medicines and Health Products (FAMHP; https://www.fagg-afmps.be/nl/fagg) which funds Farmaka asked for a new and comprehensive evaluation of its ADS. For this reason we performed a pragmatic cluster randomized controlled trial (cRCT; registered at clinicaltrials.gov (NCT01761864)). The ADS provided by FARMAKA significantly improved the proportion of recommended non-steroidal anti-inflammatory drugs (NSAIDs) prescribed by GPs but an impact on overall prescription rates of analgesics and NSAIDs was not detected. A detailed account of this is described elsewhere [16].

An understanding of academic detailers’ and GP’ views and experiences of their AD visits, and of how they present and use this information in their daily practice may help to identify the ‘active ingredients’ of this CME strategy in order to help refine and improve its implementation.

Therefore, the aim of this study was to identify reported barriers and success factors to improving AD and the quality of analgesic use for chronic pain in osteoarthritis by AD in general practice both from the perspective of the academic detailers delivering the visits and the GPs receiving them.

## Methods

This was a qualitative process study with semi-structured interviews carried out with AD and GPs who participated in a cRCT.

### The Farmaka ADS

#### Development of the content

As usual, a syllabus was developed on the basis of a systematic literature review. It contained scientific background on the topic, information on important studies and practice recommendations. Its content was evaluated by external experts. The evidence-based key messages for practice improvement were evaluated by a scientific steering group. These were translated into a presentation and leaflet which could be used as a summary fact sheet and visual aid during the visit as well as a memento or prescribing aid for the GPs that were visited. All documents are available in Dutch and French and are online available on the Farmaka website (https://www.farmaka.be/nl/artsenbezoek).

#### Key messages

The literature review suggested that paracetamol is the first choice analgesic for (chronic) pain (in osteoarthritis). If ineffective the NSAIDs ibuprofen and, in case of cardiovascular morbidity, naproxen were recommended, as is gastroprotection with proton pump inhibitors (PPI; in certain risk groups), instead of other NSAIDS, coxibs, piroxicams or other analgesics.

#### Training of academic detailers

The AD received a training on the principles of EBM. Additionally, they received a 4 days training and a syllabus which consisted of education in the scientific background of the topic as well as a training in communication skills. Practical exercises, including role playing, were given on how to present the key messages and scientific content of the visit, and to enhance the academic detailers’ communication skills.

#### Visits

During the 36- week period of the cRCT, GP practices were offered a (free) 15–20 min visit by an AD who provided four key messages but also paid special attention to the interests and specific questions of each individual GP. The summary fact sheet was explained and left behind as a reminder for the GP(s). During the visiting period, 2 days of revision were planned with the academic detailers, with further scientific discussion and training in communication skills and EBM if needed. For the cRCT, academic detailers only invited GPs from practices allocated to the intervention group for an AD visit on analgesic use for chronic pain in osteoarthritis in 2013. In 2014, practices that were allocated to the control group could also receive the same visits. For this process evaluation alongside the trial we recruited GPs from the latter practices who received such a visit.

### Study population

Among the GPs who received an AD visit, we purposively sampled 20 GPs to obtain a range of GPs from the different regions in Belgium. GPs were invited to participate in the study by the academic detailer and all were asked to participate until we had sufficient participants. If a GP agreed to participate they were telephoned by a researcher.

All 17 academic detailers who were involved in the visits on this topic were asked to participate in an interview, however only 13 were available at the time of interviewing. Participants were unaware of the trial results at the time of interview. The ethics committee of the University of Antwerp – Antwerp University Hospital granted ethics approval for the study (B300201317018).

### Study design

We interviewed participants face-to-face in their own practice or at an agreed location after informing them about the study, for example advising them that all interview data would remain confidential and anonymous, and receiving written consent to take part in the study. Interviews followed a semi-structured interview guide, which was developed collaboratively by the team and then translated into French. Interview questions asked how GPs experienced their AD visits, how they integrated the information into their consultations and what was most helpful for them in caring for their patients. The academic detailers were asked how they experienced these visits, how they handled these visits, what worked well and not so well for them and how they could improve these visits. The two experienced interviewers had a meeting prior to the interviews to standardize the interview approach. The interviews were conducted in the respective languages. The interviewers were familiar with the intervention content, although they had not designed the intervention themselves. All interviews were digitally recorded and transcribed verbatim.

### Analysis

We used inductive thematic analysis [17–19]. During the first stage the researcher (SA) read the dataset recursively to develop a preliminary coding structure. SA identified segments of text related to the research question and labelled these to create initial codes. She renamed and refined codes as further transcripts were coded. In the following stage she examined codes for similarities and differences and grouped codes accordingly to create categories and a thematic framework. She added a description of each theme and sub-theme, along with quotes to support each. In order to ensure the clarity of the themes, the research team, interviewers (SA and O.S) and the project leader of Farmaka discussed, refined and confirmed this coding structure. NVIVO10 was used to facilitate coding.

## Results

### Participants characteristics

#### Academic detailers

13 academic detailers who were available for interviewing participated in the interview. 12 of these were female and one male. The educational background of the AD ranges from general physician [4], biomedical sciences [3] and pharmacists [6] with ages ranging from 32 years to 60 years.

### General practitioners

We had to invite 34 GPs in order to have 20 GPs participating in interviews lasting from 30 min to 65 min. Of these, 12 were male and 8 female with ages ranging from 32 years to 60 years. They had two to three AD visit per year. Apart from one GP, they all also received visitors from the pharmaceutical industry with a frequency ranging from once a week to three to four visitors per working day.

### Qualitative findings

#### Academic detailers

*Theme 1: Professional role of the academic detailer: Informing* versus *actively working towards changing prescribing behavior*

The ADs view their role as providing an accurate, up-to-date synthesis of relevant information on a particular topic in a balanced and preferably engaging way. They are motivated to inform the GPs about evidence-based suggestions and to have a good discussion about these. ADs put a lot of emphasis on informing GPs, making the evidence available to them and educating them in how to interpret the evidence. They acknowledge the fact that the experience of the GP is also part of the process and should not be neglected or contested.

Encouraging a culture of critical thinking, they inform GPs of uncertainties and controversies in the interpretation of the evidence. ADs experience a lack of motivation, confidence and skills to actually motivate GPs to change their prescribing behavior or to discuss this. They view the freedom of the GPs to manage their patients as a basic principle and that is also how they want to differentiate themselves from the many commercial visitors GPs receive and who want ‘to sell’ a particular drug or convince GPs to prescribe that drug.

*“To me, it is very important that I can provide the GP with information that he or she finds useful, without making it obligatory for the GP to do something with it. If I were to say:“You have to do it like this and no you can’t do that”, I wouldn’t like it all. So the fact that the GP can do whatever he or she wants with the information is a very important element for me…I also state clearly at the beginning of the presentation that it is definitely not my aim to tell you what to do, but that we as academic detailers are here to inform you in the best possible way that we can.”(OAB2)*

*“Well, firstly we do not have a commercial function ... for most doctors, I think that is an advantage. And then, what also makes a big difference between our job and a commercial function is that we work by pathology. We give them an overview of the therapeutic possibilities with the advantages and disadvantages, and so on ... and then the doctors can make up their mind what they want to do with that information according to the data that has been presented to them”. (OAB6)*

*Theme 2: The choice of topic can impact the way academic detailers convey the message*

The ADs’ personal view on the topic might influence how they give their presentation. They prefer to give presentations on innovative topics and on new medication. They want to give advice on easy steps to implement in practice and where they expect no resistance from the GPs. They find subjects like insomnia difficult. With the present topic on NSAID they have the perception that the information is not new to the GPs. They do not like topics where they are telling GPs what they cannot do, but prefer instead to give positive informative messages that enable GPs or that provide them with a balanced choice between different types of drugs.

*“The topic of arthritis is not a theme that is top of my list of preferred topics. (…)I prefer to do more up to date topics, a topic that has more life to it. I also have the feeling that everybody already knew that giving paracetamol was the first step. To me it also felt, that the whole presentation was a bit ‘patronizing’, this is the first step, be careful with NSAIDs, they are also not ideal, careful with that…” (OAB1)*

*Theme 3: Ongoing and trusting relationship between academic detailer and GP*

Academic detailers’ think it is important to build up a good relationship with the GPs. The first visit is always seen as a difficult, but important visit to set the tone and gain trust. Further visits become easier. They are not eager to reduce the number of visits per year to their population of GPs, as they fear this could have an impact on the relationship they have built. Having to recruit new GPs is a difficult task and can prove to be an obstacle. According to the ADs, GPs who are already receiving ADs are more willing to allocate more time in their schedule to see them.

*“I like it when I know the GPs well, when I have visited them before. I do not like it at all when I do not know them ... I don’t like to call them, then going to see them, selling yourself, , canvassing, that's something I do not like at all. Once you have established a relationship it is much more pleasant.” (OAB3)*

The delivery style of the presentation depends on the relationship they have with the GP. The academic detailers experience being more confident if the visit can take place in a trusting relationship, if there is positive interaction with the GP, if the GP asks relevant questions and when they show interest in the topic. On the whole, academic detailers prefer to have as much interaction as possible. Some believe that it would be better to have more GPs together to increase this interaction and reflection between GPs in order to be able to go more in-depth and to learn from one another. A possible disadvantage for this group discussion would be that you would need to allocate more time than in a one-to-one session. Similarly, others view that the one-to-one discussion as an important factor to have the possibility to have that interaction as some GPs might feel intimated asking questions when their peers are present.

“*When I do a visit in a group practice and all the GPs attend the presentation, I receive a lot of questions, and you can go more in depth into the topic. Of course in a one to one interaction, you have the complete attention of one GP which is nice as well. However, it is nice when there is more interaction, but this can be more time consuming as well.”* (OAB2)

### GPs who receive an AD visit

*Theme 1: AD visits offer many advantages to GPs*

The interviewed GPs are positive towards these visits. They are an efficient and pleasant method of CME in their busy daily working lives. They view the ADs as knowledgeable professionals regardless of their educational background and regard them as equals even though they are not necessarily GPs. Some even see it as a bonus that the academic detailers have different background as this stimulates the discussion. The academic detailers appear credible and have a good overview of the entire topic, always related to primary care. This is in contrast to the many visits the GPs still receive from medical representatives of the pharmaceutical industry who only know the product they want to sell and have limited knowledge on the topic as a whole. These pharmaceutical visits are not seen as a discussion between peers. Yet, the information from the industry is also valuable to them, as it keeps the GPs up to date on the newest medications on the market. Through the AD, the GPs learn to reflect critically on the messages they receive from the pharmaceutical industry and take the evidence presented to them with a pinch of salt.

*“I receive an AD now once every three months, and for me that's really a ‘breath of fresh air, so to speak, it's a refreshing and a fun interlude in between my daily activities. They are very up to date with the issues and they present it in its completeness especially tailored to primary care. I think that is a very good thing, because when I am very busy I sometimes see up to two commercial representatives in a morning session, sometimes even more and that is not very nice at all.”(H6)*

*“I think that the greatest merit is that these visits are giving a ‘global synthesis’, a problem such as osteoarthritis is being presented as a whole, schematically presented, with easy to follow steps, all clarified, it gives you a good plan and a good balanced fact sheet with the pros and cons.” (H13*)

The participants find all primary care topics relevant as long as they are well documented and relevant to general practice as well as frequently encountered in their clinical care. They find it invaluable to discuss topics that they know well to make them reflect on their embedded routine behavior.

*“Yes it is useful because the topic of osteoarthritis or other topics that they have presented, they are very common problems in our practice and the presentations are basics and it is always useful that we GPs are being reminded of the basics again. By force we work by habit…” (H11)*

*Theme 2: Impact on daily practice in relation to the topic of arthrosis, old habits die hard*

The GPs feel confident about their management of arthrosis and admit that they do not often reflect on their own prescribing behavior. They do find the structured suggestions in the presentation easy to apply in daily practice, but acknowledge that GPs always need to balance out and discuss with their patient what is achievable or acceptable for them. For the GPs, the presentation includes both new and relevant information and it clarifies some earlier misconceptions, especially about the safety issues of analgesics. Some GPs notice that they have not yet experienced any difficulties yet in their practice. However, they are eager to use that information and find the explanation of the risks of certain medication useful. When contrasting their own prescribing behavior with the EBM recommendations, the mismatches trigger them to reflect on it. However, they acknowledge that actively changing behavior is not that easy and straightforward. Having the information is one thing. In order to adapt to it, GPs need the necessary tools.

*“I think I still use a lot of anti-inflammatories and that I put too little emphasis on regular analgesia, physiotherapists, good mobilization, weight, avoid overload, and so on. These are matters which I partially knew, but the information on anti-inflammatories were of course an 'eye-opener' to me, of course, they usually lead to patient satisfaction, mostly anyway, but I will still try to go easier on them .... You will recall certain things which it is good and you will see confirmation of things that you already thought you knew or things that you didn’t know. The topic of osteoarthritis was actually good.” (H6)*

*“Sometimes I do think, strange I have never really encountered this in my practice, or the presentation seems different from my management, and then I do not find it easy to change my behavior. Because I do not always experience these disadvantages, and that is a difficult one, because on the one hand, I really want to do well, but on the other hand, it is going well the way I am doing it now, why would I change my behavior. It is always difficult to balance that out (…)But I do think, one thing that has stuck and I will change this immediately with new patients, is that I will be a lot more restrained with prescribing NSAIDs, that I have definitely picked that up from the presentation.” (H3)*

*Theme 3 Suggestions to optimize visits*

Overall, GPs are very positive and keen towards the visits. If possible they would prefer more visits as these are an easy and pleasant way to keep up to date with good practice. The GPs support the views of the academic detailers that the relationship between the academic detailer and the GP is built on trust and the frequency of the AD visits, and that a good relationship as well as group discussions enhances the interaction between GPs and academic detailers, therefore creating more in-depth discussions. Interactions with colleagues can enhance the peer learning as well as the reflection process. Stimulating questions or reflections from peers on how they handle or encounter these issues in day to day practice can enhance the discussion with the AD. The leaflet can act as and this is appreciated by the GPs as it is concise and up to date. However, they admit that they rarely look at it again and that it is often buried under the usual piles of paper. Yet, they have no suggestions for improvement as they like the format. GPs find it very practical that at the end of the session, a future appointment is scheduled in their diaries.

*“Each time the presentations become more interactive, because luckily enough it is always the same lady, we are starting to get to know each other a bit better, and then automatically the session becomes more interactive. In the beginning my colleague and I were just good listeners; we were both just sitting quietly. She then asked whether we had any questions, but we didn’t really dare to ask any questions. That has completely changed now, if she explains something, and I am not sure whether i have understood it well, or i have a different idea about it I will ask for more explanation. She has always presented it in an interactive way, but I think you need to have that trusting relationship first before you can have a good interaction. I very much appreciate it that it is always the same person.” (H2)*

*“It would be good not to have just individual sessions, but also group sessions, because in my experience, you generate more questions from group discussions, you create a certain dynamic, now i sometimes feel I am a bit stuck on my chair by myself, I would prefer and I also think it is an essential element to have that group dynamic.” (H4)*

## Discussion

In general, GPs view AD visits as a practical and useful CME approach in primary care, that are less time consuming than other CME activities. GPs make a clear distinction between academic detailers and representatives of the pharmaceutical industry. They find them both useful, but with a different goal. Academic detailers are seen as equals and credible agents, even though they might have a different educational background. They bring an overview of objective and independent information relevant to their daily practice and with whom they can have a discussion. By comparison, the pharmaceutical industry gives them the latest information on the newest medicine on the market but with the aim to ‘sell’ the product.

Academic detailer, see themselves as content experts and their main job is to inform GPs about the subject. There is no emphasis on the process of change. Both GPs and academic detailers believe that the visits might have more interaction and discussion if they would be in smallish groups. However, they like the one-to-one interactions and find it easier to schedule the visits into their diaries if it takes place in their practice. The visits provide an opportunity to address GPs’ needs in a timely way, relevant to the context in which GPs are providing care. The osteoarthritis topic provided the GPs with some new and relevant information and clarified some misconceptions. They have increased awareness of the disadvantages of particular NSAIDs and of the limited doses of paracetamol that should be prescribed for chronic use. It may have changed their beliefs and/or attitudes towards more appropriate analgesic prescribing for osteoarthritis. However, the GPs acknowledge that they do lack the confidence or the tools to manage their patients differently. The transfer of knowledge into practice is not so straightforward.

### Comparison to the literature

To date the AD visits have been delivered as a stand-alone intervention. However, it has been shown that a multifaceted strategy may increase the likelihood that efforts to improve in GP practice will be successful [2]. It was interesting to find that both GPs and academic detailers preferred (more) interaction, since there is evidence from systematic reviews that CME activities are effective for improving GPs performance and patient outcomes if they are highly interactive and involve multiple exposures [7]. In a recent systematic review, the authors state that the most effective interventions call for coherence but also emphasize collective action and reflexive monitoring. These interventions provide mechanisms for participants to relate their performance to external reference group expectations, and create opportunities for reinforcing internal peer group norms [20]. Therefore, feedback about their prescribing behavior in relation to their peers might optimize the intervention and also stimulate the discussion [19, 20].

Habraken et al. found that Belgian physicians highly rated AD visits. Approximately 90% of those who used the ADS wished to use it again [11], which is in accordance with our findings. GPs were eager to schedule new appointments and would rather have more visits than a decline in the number of visits. Habraken et al. also identified the following barriers to participation: the information was not new or could be obtained in other ways, the information was politically colored and designed to cut expenses, and the educational visits were time-consuming [11, 15]. Also in the study by Allen M, GPs did not see AD as efficient and convenient [21]. Our findings suggested that AD visits are perceived as an efficient and convenient way of participating in CME. The educational background of the AD seems appropriate and is valued. Yet, in addition to the content expertise they have, the process expertise – knowing how to effect behavior changes and knowing how to communicate information in ways that promote evidence-based care – seems to be lacking [22, 23] and is reflected in the RCT results [16]. Special training might be critical to improve the expected outcomes.

### Strengths and limitations of this study

This study provides a valuable insight into GPs’ and academic detailers’ perceptions of their AD visits. It is a strength of the study that the views of the GPs and ADs can be compared.

All academic detailers were interviewed. The sampling of GPs was restricted by the resources of this study and sampling in the intervention arms was prioritized. As a result GPs who were not willing to receive an AD visit, were not interviewed.

Academic detailers contacted the GPs themselves to ask for their agreement to take part in the study. These GPs are probably more favorable towards AD than the general population of GPs. Our findings should be interpreted in the light of this possible bias. However, we were encouraged to find that all participants in this study freely reported their negative views on aspect of the visits, indicating that they were comfortable critiquing or giving feedback about their AD visit and Farmaka’s ADS.

### Implication for research and practice

Overall these findings suggest that the visits of the academic detailers on the topic of NSAIDs are a well-accepted intervention. It is an appreciated way of independent CME and a counterbalance to the frequent visits of the pharmaceutical industry. Individual contacts are valuable, but peer interaction in a small group could enhance discussion and increase reflection on their prescribing behavior. At the time of the interviews, the ADs were not actively working towards changing behavior and were using the visits to inform the clinicians. In addition to content expertise, they should be trained in knowing how to effect behavior change, i.e. communicate information in ways that is more than just informative. They should receive training on communication and interpersonal skills to overcome anticipated GP barriers to behavior change. Audit and feedback information could enhance reflection of GPs prescribing behavior and could be a basis of interaction and discussion. Repetition of information is necessary to achieve change in behavior, different organizations need to give similar messages, multiple exposures are necessary in order to achieve change. A low intensity approach is not likely to produce change.

## Conclusions

GPs experience AD as a credible and interesting way of CME that enhances their knowledge and makes them reflect on their prescribing behavior.

## Abbreviations

AD: Academic detailing
ADS: Academic detailing service
CME: Continuing medical education
cRCT: Cluster-randomized controlled trial
DDD: Defined daily dose
EBM: Evidence-based medicine
GP: General practitioner
GPs: General practitioners
NSAID: Non-steroidal anti-inflammatory drug
PPI: Proton pump inhibitor
RCT: Randomized controlled trial
